# Supplementary material for: MARCH8 Suppresses Tumor Metastasis and Mediates Degradation of STAT3 and CD44 in Breast Cancer Cells
Source: Cancers (Basel). 2021 May 22;13(11):2550. doi: 10.3390/cancers13112550 (PMC8196951; doi:10.3390/cancers13112550)
Supplement: Supplementary file 1 [file cancers-13-02550-s001.zip › cancers-1181715-supplementary.pdf]

Fig 3A		M8	actin	ratio	
	1	3270.983	4874.062	0.6711	1.00
	2	805.406	7441.912	0.108226	0.16
	3	442.042	7151.912	0.061808	0.09
	4	719.991	6025.418	0.119492	0.18
	5	611.184	4747.305	0.128743	0.19
	6	276.506	4674.669	0.05915	0.09

Fig 3B		M8GFP	actin	r
	1	89.364	3767.456	0.02372
	2	5794.418	3257.92	1.778564

Fig 4B		caspase	M8	actin	r caspase	r M8
	1	0	0	5822.648	0	0
	2	3079.619	0	6593.255	0.467086	0
	3	3417.698	0	5545.234	0.616331	0
	4	0	14501.95	5833.184	0	2.486111
	5	5576.598	14800.14	6098.012	0.914494	2.427043
	6	8839.548	15081.87	7625.255	1.159246	1.977885

Fig 5A		CD44	BAX	BID	M8 GFP	actin	rCD44	rBAX	rBID	rM8
	1	6914.477	705.456	2114.406	0	6618.962	1.044647	0.106581	0.319447	0
	2	740.092	3315.698	7144.548	11391.36	6821.305	0.108497	0.48608	1.047387	1.669968
			?							

Fig 5D		CD44	M8 GFP	actin	rCD44	rM8
	1	13473.95	0	11803.23	1.141548	0
	2	2422.619	13435.97	11520.23	0.210293	1.166294
	3	757.698	16158.85	9951.033	0.076143	1.623837
	4	11117.49	15158.15	9927.397	1.11988	1.5269

Fig 5F		BAX	BID	CD44	M8 GFP	actin	rBAX	rBID	rCD44	rM8
	1	0	0	14085.59	0	11198.98	0	0	1.257756	0
	2	4456.184	5317.426	7682.569	15020.71	11757.4	0.379011	0.452262	0.653424	1.277554
	3	3115.355	3088.184	11488.71	13698.39	12167.05	0.256048	0.253815	0.944248	1.125859

Fig 6A		AKT	pAKT	ERK	pERK	actin	rAKT	rpAKT	rERK	rpERK
	1	3435.983	7772.447	5498.782	7988.69	7385.962	0.465205	1.052327	0.744491	1.081605
	2	3351.912	6541.497	5655.368	9790.518	7433.962	0.450892	0.879948	0.760747	1.316999
	3	2867.548	8129.347	5978.371	7238.569	6819.426	0.420497	1.192087	0.876668	1.061463
	4	7254.033	6757.912	5421.974	7994.933	6345.962	1.143094	1.064915	0.854397	1.259846

Fig 6D		CD44 A	CD44 S	STAT A	STAT S	BID A	BID S	BAX A	BAX S	actin A	actin S
	1	9219.397	7136.79	5697.184	5730.305	0	0	0	0	7641.598	7323.891
	2	4995.669	3162.891	6904.962	7615.225	4418.113	3794.719	2805.77	4855.941	8360.548	7517.255
	3	11841.84	12129.98	7000.548	6121.083	2891.527	1990.355	1403.82	2702.77	8500.497	7562.134
	4	2634.134	3884.083	6625.255	6131.79	2489.698	1797.527	1747.698	1882.355	8622.912	7972.305
	5	3564.548	2458.012	10346.18	10092.49	1250.406	847.82	1201.82	816.163	8732.326	6897.305
	6	3820.669	1124.234	7323.447	5435.255	4386.648	3945.698	5184.527	2633.941	8523.497	7035.719

		rCD44 A	rCD44 S	rSTAT A	rSTAT S	rBID A	rBID S	rBAX A	rBAX S
	1	1.206475	0.974453	0.745549	0.782413	0	0	0	0
	2	0.597529	0.420751	0.825898	1.013033	0.528448	0.504801	0.335596	0.645973
	3	1.393077	1.604042	0.823546	0.809439	0.34016	0.2632	0.165146	0.357408
	4	0.305481	0.487197	0.768332	0.769136	0.288731	0.225471	0.202681	0.236112
	5	0.408201	0.356373	1.184813	1.463251	0.143193	0.12292	0.137629	0.118331
	6	0.448251	0.159789	0.859207	0.772523	0.514654	0.560809	0.608263	0.374367

Fig 3. MARCH8 induces apoptosis in TNBC cells

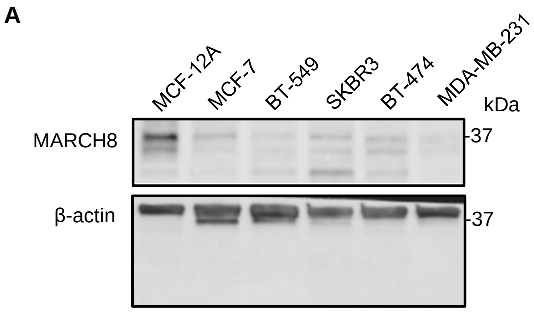

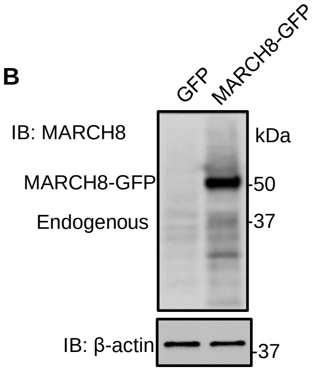

Fig 4. MARCH8 inhibits colony formation *in vitro* and lung colonization *in vivo*

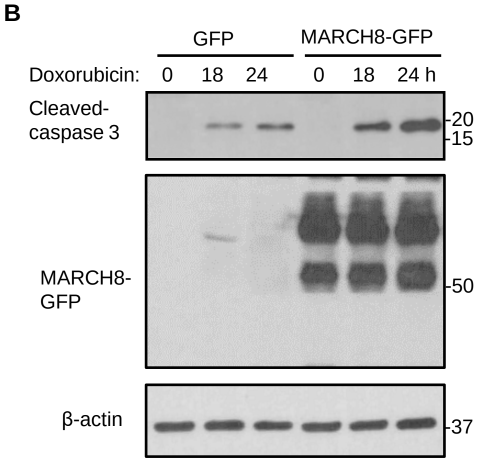

Fig 5. MARCH8 interacts with and degrades CD44 through the lysosome pathway.

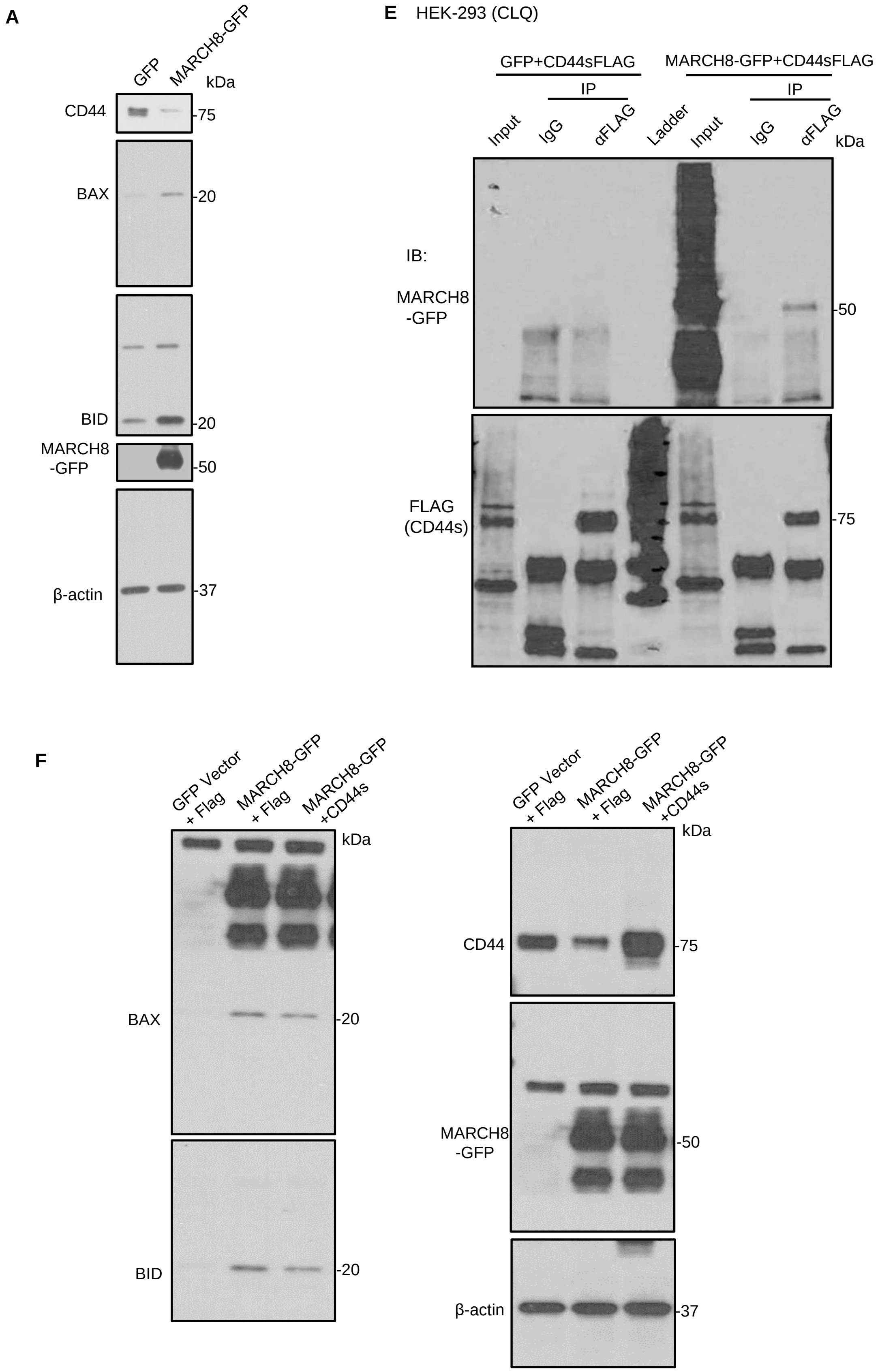

Full blot for Figure 6

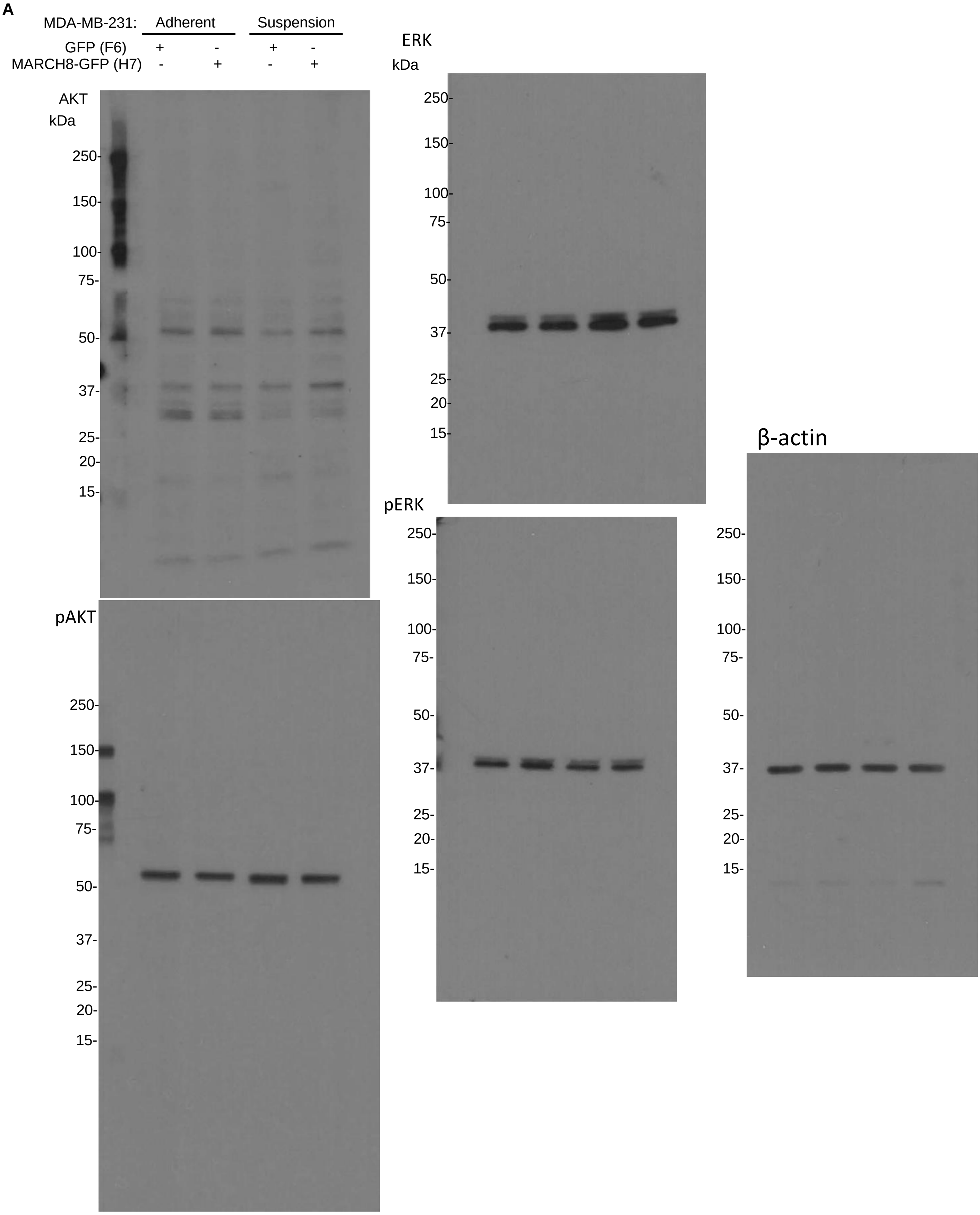

B

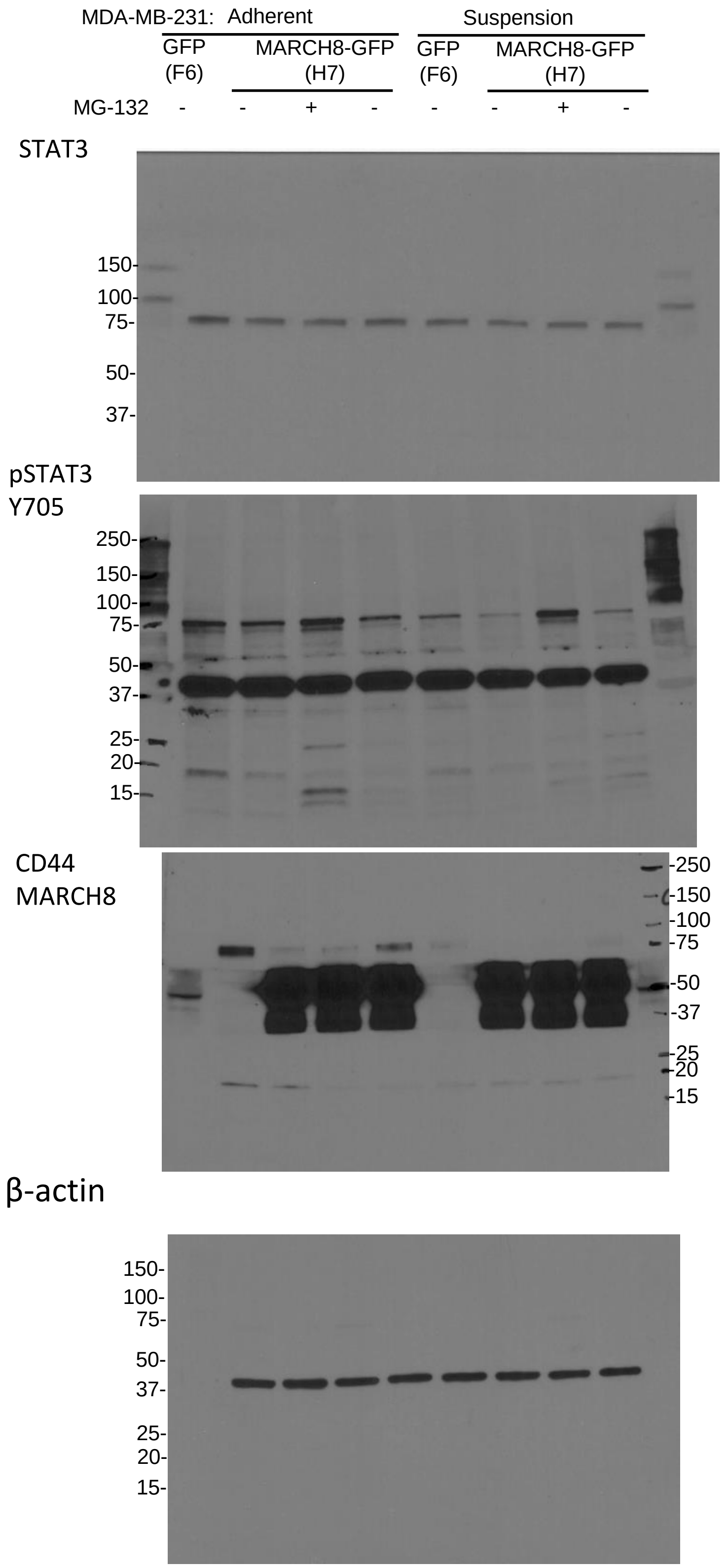
MDA-MB-231: Adherent
Suspension
GFP (F6)
MARCH8-GFP (H7)
GFP (F6)
MARCH8-GFP (H7)
MG-132 - - + - - - + -
STAT3
150-
100-
75-
50-
37-
pSTAT3 Y705
250-
150-
100-
75-
50-
37-
25-
20-
15-
CD44 MARCH8
-250
-150
-100
-75
-50
-37
-25
-20
-15
β-actin
150-
100-
75-
50-
37-
25-
20-
15-

C

HEK293 cells + MG-132:

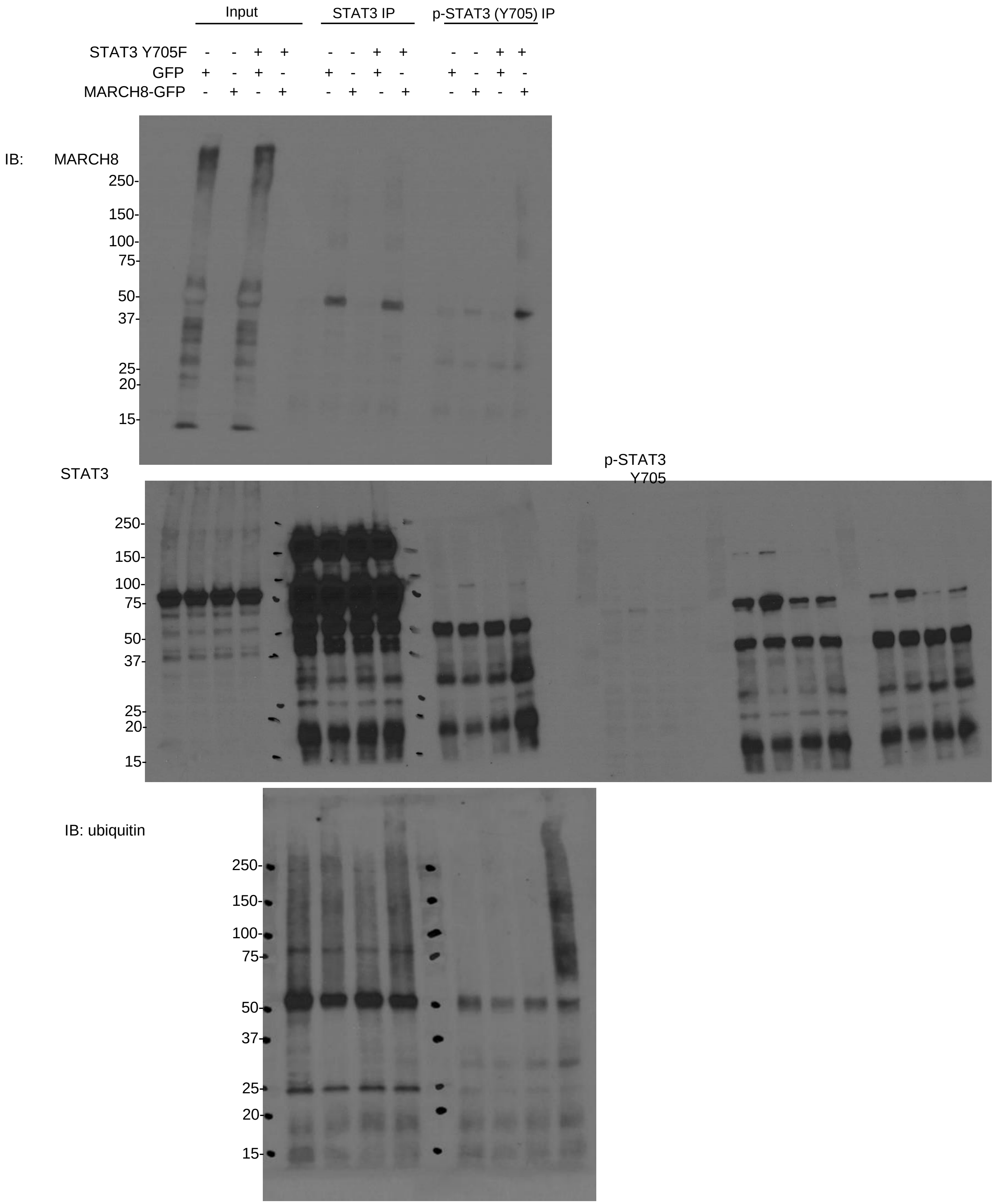

D

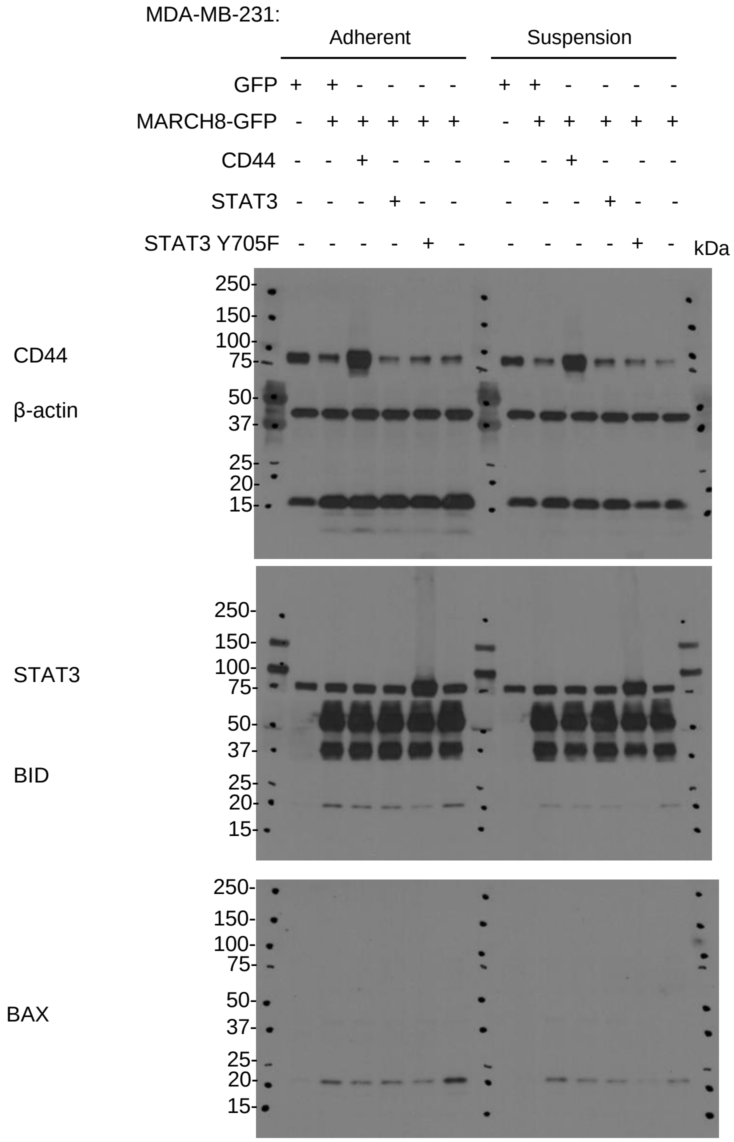
MDA-MB-231:
Adherent
Suspension
GFP
MARCH8-GFP
CD44
STAT3
STAT3 Y705F
kDa
250-
150-
100-
75-
50-
37-
25-
20-
15-
CD44
β-actin
STAT3
BID
BAX
